# Efficient production of clerodane and *ent*-kaurane diterpenes through truncated artificial pathways in *Escherichia coli*

**DOI:** 10.3762/bjoc.18.89

**Published:** 2022-07-21

**Authors:** Fang-Ru Li, Xiaoxu Lin, Qian Yang, Ning-Hua Tan, Liao-Bin Dong

**Affiliations:** 1 State Key Laboratory of Natural Medicines, School of Traditional Chinese Pharmacy, China Pharmaceutical University, Nanjing 211198, Jiangsu, China

**Keywords:** artificial pathway, *ent*-kaurene, *Escherichia coli*, overproduction, terpentetriene

## Abstract

The clerodane and *ent*-kaurane diterpenoids are two typical categories of diterpenoid natural products with complicated polycyclic carbon skeletons and significant pharmacological activities. Despite exciting advances in organic chemistry, access to these skeletons is still highly challenging. Using synthetic biology to engineer microbes provides an innovative alternative to bypass synthetic challenges. In this study, we constructed two truncated artificial pathways to efficiently produce terpentetriene and *ent*-kaurene, two representative clerodane and *ent*-kaurane diterpenes, in *Escherichia coli*. Both pathways depend on the exogenous addition of isoprenoid alcohol to reinforce the supply of IPP and DMAPP via two sequential phosphorylation reactions. Optimization of these constructs provided terpentetriene and *ent*-kaurene titers of 66 ± 4 mg/L and 113 ± 7 mg/L, respectively, in shake-flask fermentation. The truncated pathways to overproduce clerodane and *ent*-kaurane skeletons outlined here may provide an attractive route to prepare other privileged diterpene scaffolds.

## Introduction

Diterpenoids, of which there are over 34,000 members (http://terokit.qmclab.com), have attracted great attention from chemists and biologists due to their intriguing chemical structures and broad pharmacological functions [1–4]. The vast structural diversity of diterpenoids arise biosynthetically from the following two stages: i) diterpene synthase (DTS, also called diterpene cyclase) act on geranylgeranyl diphosphate (GGDP) to perform regio- and stereoselective cyclizations or skeleton rearrangement reactions via carbocation chemistry to form diverse and versatile carbon skeletons; and ii) multiple post-modification enzymes, most often cytochrome P450s, decorate the carbon skeletons resulting in a large array of oxidative diversity [5–7]. Nature’s ability to efficiently biosynthesize diterpenoids has attracted chemists to mimic it for the synthesis of complex diterpenoids using either pure chemical tools, exemplified by the ‘two-phase strategy’ pioneered by the Baran group or a combination of enzymatic and chemical tools (chemoenzymatic synthesis) [8–11]. Despite great efforts spanning several decades, de novo organic synthetic methods access to the core diterpene skeletons are still highly challenging owing to their numerous chiral centers and polycyclic complexity [12]. Additionally, chemical transformations from commercial natural products are also tedious and currently limited to a few diterpene skeletons [8].

**Figure 1:**
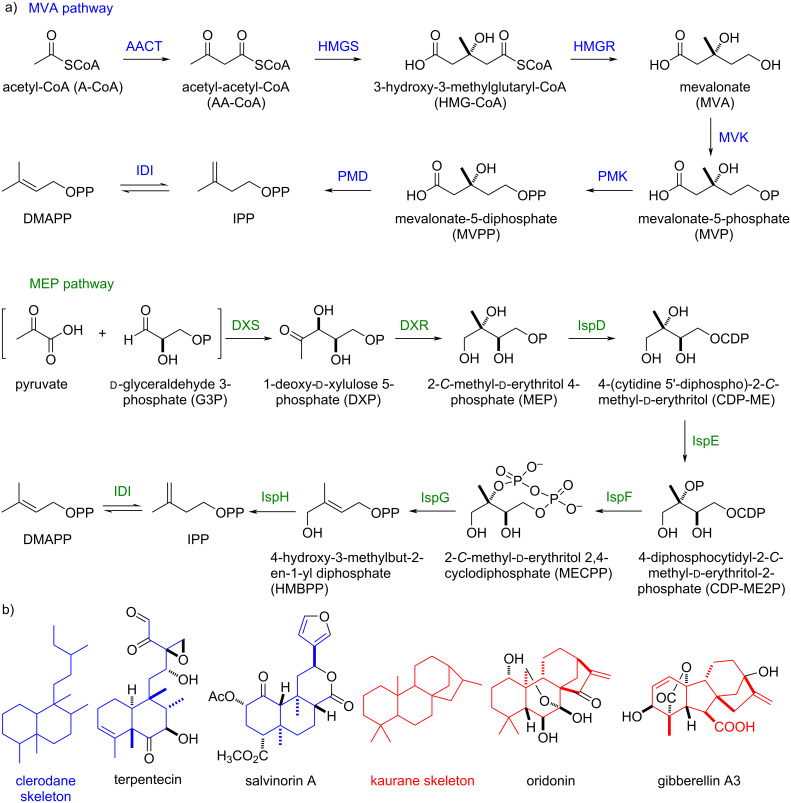
(a) The natural pathways (MVA: blue, MEP: green) for producing IPP and DMAPP; (b) the carbon skeletons of clerodane and kaurane diterpenes and representative bioactive natural products. acetoacetyl-CoA thiolase (AACT); HMG-CoA synthase (HMGS); HMG-CoA reductase (HMGR); mevalonate kinase (MVK); phosphomevalonate kinase (PMK); diphosphomevalonate decarboxylase (PMD); 1-deoxy-ᴅ-xylulose 5-phosphate synthase (DXS); 1-deoxy-ᴅ-xylulose 5-phosphate reductoisomerase (DXR).

Engineering microbes via synthetic biology provides new opportunities to produce terpenoid carbon skeletons. All terpenoids are derived from the minimum C_5_ isoprenoid building blocks isopentenyl diphosphate (IPP) and dimethylallyl diphosphate (DMAPP), which are produced in the cell via one of two pathways: i) the mevalonate (MVA) pathway includes seven steps from acetyl-CoA (A-CoA); and ii) the 2-*C*-methyl-ᴅ-erythritol 4-phosphate (MEP) pathway includes eight steps starting from the condensation of pyruvate and ᴅ-glyceraldehyde 3-phosphate (G3P) [13–14] (Figure 1a). Due to these lengthy biosynthetic steps as well as complex metabolic regulations and extensive cofactor requirements, several groups have engineered elegant bypass pathways to mitigate pressures on gatekeeper enzymes [15–18]. However, these systems are still dependent on the entry points within the MVA or MEP pathways [17–18]. Recently, the Stephanopoulos and Williams groups reported two-step artificial pathways to efficiently produce isoprenoid precursors IPP and DMAPP from isopentenol (ISO) and dimethylallyl alcohol (DMAA) [15,19]. In this strategy, two independent kinases were used. ISO and DMAA were phosphorylated to form isopentenyl monophosphate (IP) and dimethylallyl monophosphate (DMAP), respectively, which were then phosphorylated by another kinase to produce IPP and DMAPP [19–21]. Notably, this pathway successfully bypassed the limitations of native isoprenoid biosynthetic pathways, resulting in the overproduction of multiple (mero)terpenoids such as lycopene, *cis*-abienol, and prenylated tryptophan [15,19,22–23].

The clerodane and *ent*-kaurane diterpenoids are two categories of diterpenoids that are widely distributed in terrestrial plants, fungi, and a few bacteria and possess broad pharmacological bioactivities [1–3]. Representative natural products containing these skeletons are terpentecin (cytotoxic and antibiotic), salvinorin A (kappa opioid receptor), oridonin (cytotoxic), and gibberellin (phytohormone) (Figure 1b) [24–27]. How to efficiently construct the core carbon skeletons is a critical question in utilizing the advanced ‘two-phase strategy’ or chemoenzymatic synthesis to readily synthesize clerodane and *ent*-kaurane diterpenoids. In this paper, we report the reconstruction of truncated artificial pathways to overproduce two representative clerodane and *ent*-kaurane diterpenes, terpentetriene and *ent*-kaurene, in *E. coli*. The titers of terpentetriene and *ent*-kaurene were optimized to 66 ± 4 mg/L and 113 ± 7 mg/L, respectively, in shake-flask fermentation.

## Results and Discussion

### Constructing a two-step artificial pathway to overproduce IPP and DMAPP precursors

Following the Williams design, *phoN* and *ipk* from *Shigella flexneri* and *Thermoplasma acidophilum*, respectively, were codon-optimized and synthesized for overexpression in *E. coli* [19]. Isopentenyl diphosphate isomerase (IDI) from *E. coli,* which balances IPP and DMAPP in vivo, was also included in our construct. To initially test the efficiency of this two-step artificial pathway, we constructed strains DL10001 (*phoN*, *ipk* and *idi* plus the lycopene-producing genes *crtE*, *crtB*, and *crtI*) and DL10002 (only *crtE*, *crtB*, *crtI*, and *idi*) [28]. Compared to strain DL10002, DL10001 produced significantly larger amounts of lycopene after feeding 6 mM ISO and DMAA (3:1) in a 3-day fermentation (Figure S1, Supporting Information File 1). This result supported that our reconstructed two-step artificial pathway efficiently produced IPP and DMAPP and thus can be used to overproduce the clerodane and *ent*-kaurane diterpenes in *E. coli*.

### Collecting the essential genes in the biosynthesis of terpentetriene and *ent*-kaurene

**Figure 2:**
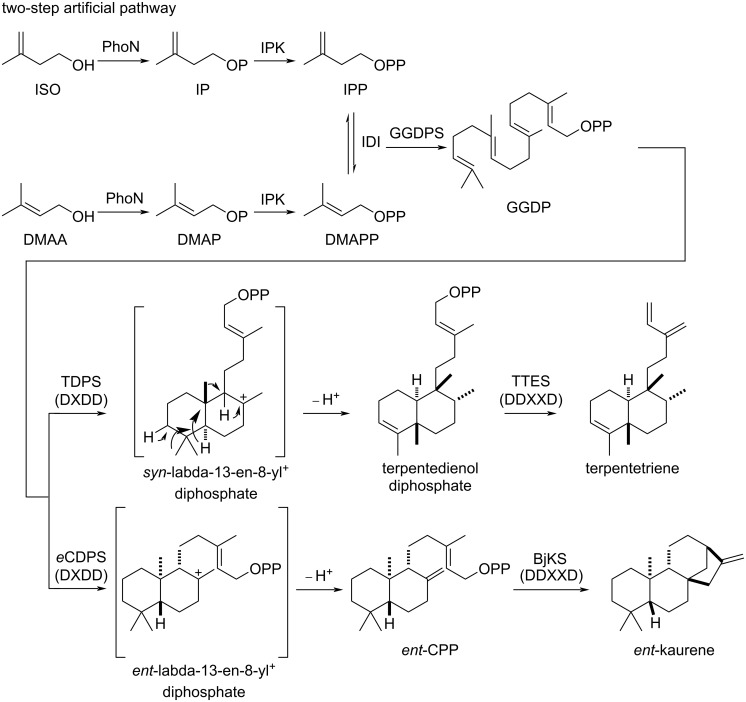
Truncated artificial pathways (six steps) to produce terpentetriene and *ent*-kaurene.

Terpentetriene and *ent*-kaurene are labdane-related diterpenes and biosynthetically constructed by two sequential DTSs from the common C_20_ linear allylic diphosphate GGDP [29]. Terpentetriene was the proposed biosynthetic intermediate of terpentecin, an anticancer and antibiotic natural product isolated from *Kitasatospora griseolosporeus* MF730-N6 in 1985 [24,30]. In the biosynthesis of terpentetriene, GGDP was first cyclized by a class II DTS (Cyc1) that contains a conserved DxDD motif to form terpentedienyl diphosphate (TDP) via a *syn*-labda-13-en-8-yl^+^ diphosphate intermediate (Figure 2), which, prior to deprotonation, can be followed by rearrangement to form the clerodane skeleton. TDP was then ionized by a class I DTS (Cyc2) that contains a conserved DDxxD motif and through a deprotonation to install a terminal double bond at the side chain [31–32]. We were unable to access the original terpentetriene producing strain of *K. griseolosporeus* MF730-N6, as well as two possible producers (*Streptomyces* sp. San01 and *Kitasatospora* sp. CB02891) after a survey of the existing genome sequence databases, however, we discovered a strain, *Kitasatosporia griseola* DSM 43859, without a genome sequence disclosed, from the strain library of CGMCC. Using the primers designed from the sequences of *cyc1* and *cyc2*, we fortunately obtained two genes of expected lengths, which we named *tdps* and *ttes*, by polymerase chain reaction (PCR). The sequencing results of *tdps* and *ttes* showed extremely high identities with those of *cyc1* and *cyc2* (97% and 99%, respectively) (Table S4, Supporting Information File 1). Additionally, a GGDP synthase named GGDPS was also successfully cloned from the same strain. These results suggested that *K. griseola* DSM 43859 is a different strain with *K. griseolosporeus* MF730-N6 and likely includes a similar biosynthetic gene cluster in the production of terpentetriene. The medium screening for terpentetriene production as well as the elucidation of terpentetriene biosynthetic pathway in *K. griseola* DSM 43859 are underway in our lab. Thus, all essential genes (*phoN*, *ipk*, *idi*, *ggdps*, *tdps*, and *ttes*) for a truncated artificial pathway to produce the clerodane diterpene, terpentetriene, were fully collected.

The biosynthetic pathway towards *ent*-kaurene resembles that of terpentetriene. First, a class II DTS catalyzes the cyclization of GGDP into a diphosphate intermediate, *ent*-copalyl diphosphate (*ent*-CPP). Next, a class I DTS further cyclizes *ent*-CPP into the target tetracyclic skeleton, *ent*-kaurene (Figure 2). In this study, the *ent*-CPP synthase (*e*CDPS) gene was cloned from *Streptomyces* sp. NRRL S-1813, which was an alternative *ent*-kaurenol-derived antibiotic platensimycin producer [33–35], while the *ent*-kaurene synthase (BjKS) gene was from *Bradyrhizobium japonicum*, a bacterial symbiont of soybean that is known to produce the *ent*-kaurene-derived phytohormone gibberellin [36–37]. The *bjks* gene was codon-optimized and synthesized for overexpression in *E. coli*. We used the same *ggdps* from *K. griseola* DSM 43859 in this construct. Thus, the full pathway to *ent*-kaurene, possessing six genes (*phoN*, *ipk*, *idi*, *ggdps*, *ecdps*, and *bjks*), was completed.

### Constructing truncated artificial pathways to produce terpentetriene and *ent*-kaurene

**Figure 3:**
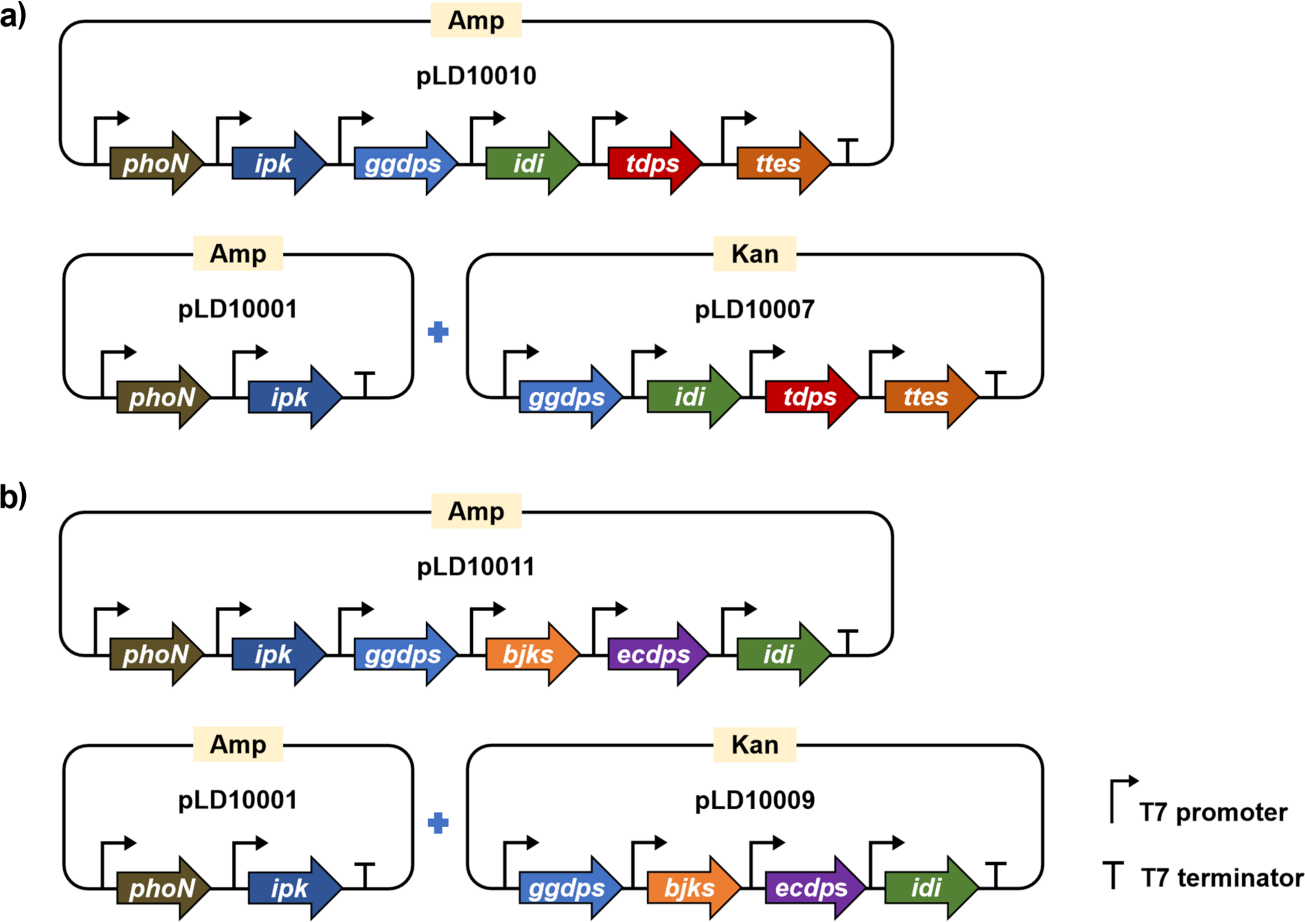
Construction maps of single plasmid expression system and two-plasmid expression system for overproducing terpentetriene (a) and *ent*-kaurene (b) in *E. coli*.

After collecting all the essential genes, we initially designed two different expression systems for producing terpentetriene and *ent*-kaurene from ISO and DMAA (Figure 3). To decrease the burden on the host cell [38], the six genes (*phoN*, *ipk*, *idi*, *ggdps*, *tdps*, and *ttes*) leading to the production of terpentetriene, each with a strong and inducible T7 promoter, were cloned into pETDuet-1 plasmid to form pLD10010, which was transformed into *E. coli* BL21 (DE3) to create strain DL10003. In another expression system, the upstream two genes, *phoN* and *ipk*, were cloned into pETDuet-1 plasmid, while the other four genes (*idi*, *ggdps*, *tdps*, and *ttes*) were cloned into pRSFDuet-1 plasmid. Both plasmids were co-transformed into *E. coli* BL21 (DE3) to create strain DL10004. Using the same protocol, another two strains, DL10005 with a single plasmid expression system and DL10006 with a two-plasmid expression system, that included the whole truncated artificial pathway (*phoN*, *ipk*, *idi*, *ggdps*, *ecdps*, and *bjks*) for *ent*-kaurene production were created.

Next, DL10003–10006 were fermented in Lysogeny broth (LB) medium supplied with 1% glycerol, 6 mM ISO/DMAA 3:1, and 0.1 mM isopropyl β-ᴅ-1-thiogalactopyranoside (IPTG) inducer. In comparison with the negative control of wild-type *E. coli* BL21 (DE3), all strains successfully produced new peaks in the HPLC profiles after a 3-day fermentation. Larger scale (3 L) fermentations of DL10004 and DL10006 led to the isolation of 45 mg and 90 mg of terpentetriene and *ent*-kaurene, respectively, whose ^1^H and ^13^C NMR spectra supported their chemical structures (Figures S4–S7 in Supporting Information File 1) [31]. These results demonstrated that the two-step artificial pathway coupled with downstream genes for the terpentetriene and *ent*-kaurene biosynthesis was successful and efficient. Though a single plasmid expression system might lower the host cell burden, our results showed that the two-plasmid expression system produced significantly more amounts (14-fold) of terpentetriene and (3-fold) of *ent*-kaurene than single plasmid expression system (22 ± 4 mg/L vs 1.6 ± 0.2 mg/L for terpentetriene, and 27 ± 3 mg/L vs 8.1 ± 0.2 mg/L for *ent*-kaurene). DL10004 and DL10006 were therefore selected for subsequent fermentation optimization.

### Optimizing the ISO/DMAA concentrations

**Figure 4:**
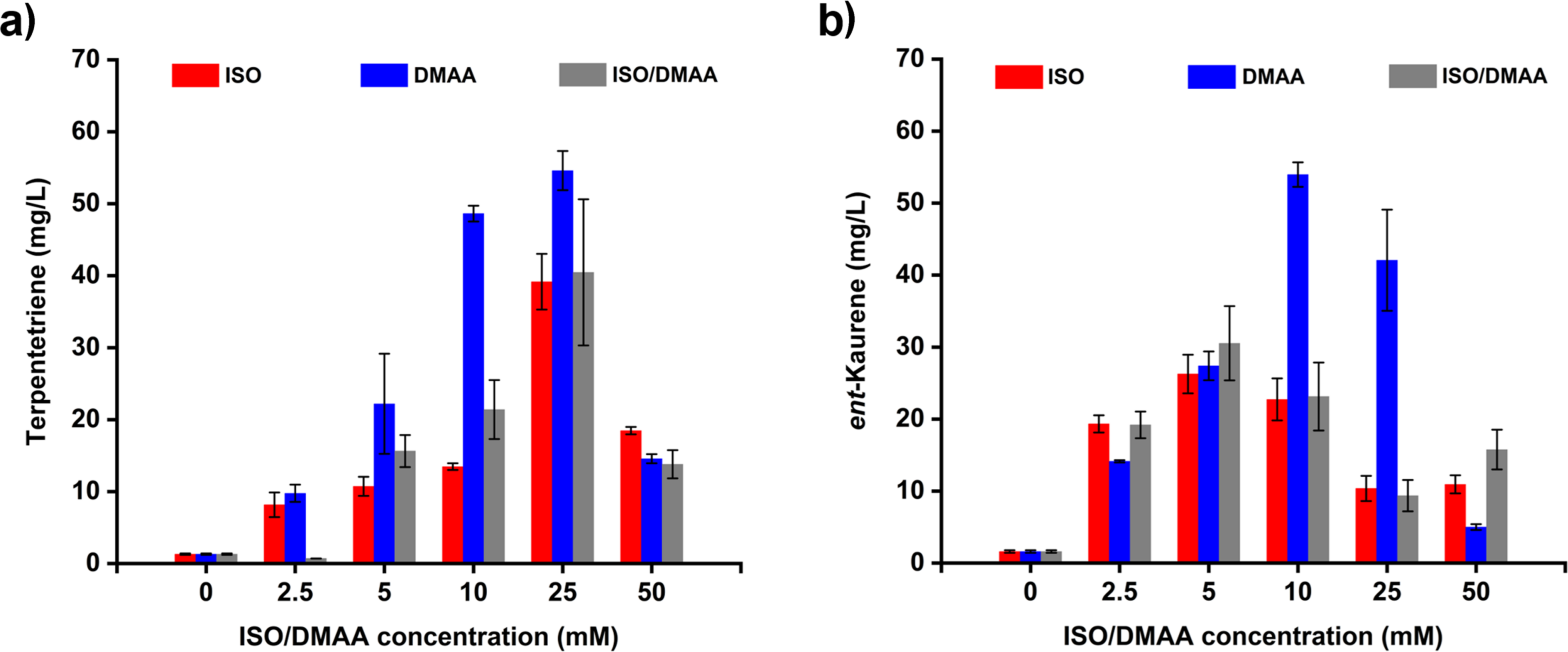
Optimizing the ratios of ISO/DMAA for overproducing terpentetriene (a) and *ent*-kaurene (b). Red: ISO; blue: DMAA; gray: ISO/DMAA 3:1 mixture. All product yields are reported as means ± SD of three replicates.

To determine the ideal concentrations of exogenous supplementary ISO/DMAA for producing terpentetriene and *ent*-kaurene, a series of feeding experiments was carried out with various concentrations of ISO/DMAA. When strains DL10004 and DL10006 that were cultured in LB medium reached an OD_600_ of 0.6, 0.1 mM IPTG and different amounts of ISO/DMAA were added. After 3-day fermentations in the absence of ISO/DMAA, both strains only produced small amounts of terpentetriene and *ent*-kaurene (1.3 ± 0.1 mg/L and 1.6 ± 0.2 mg/L, respectively), suggesting a low expression level of the endogenous MEP pathway in *E. coli* (Figure 4). When ISO, DMAA, or a mixture of ISO/DMAA 3:1 were exogenously added, the yields of terpentetriene and *ent*-kaurene increased dramatically. The highest yields of terpentetriene (55 ± 3 mg/L) and *ent*-kaurene (53 ± 2 mg/L) were observed in the presence of 25 and 10 mM of DMAA, respectively. These results demonstrated that i) the introduced two-step artificial pathway could efficiently convert exogenous supplemented ISO and DMAA into a pool of hemiterpenes; and ii) IDI effectively balances the proportion of IPP and DMAPP. Given that the commercial DMAA is cheaper than ISO, our result in merely using DMAA should be helpful in decreasing the overall cost for the production of other terpenes using this two-step artificial pathway [22].

### Optimizing the concentrations of glycerol and IPTG, and fermentation time course

**Figure 5:**
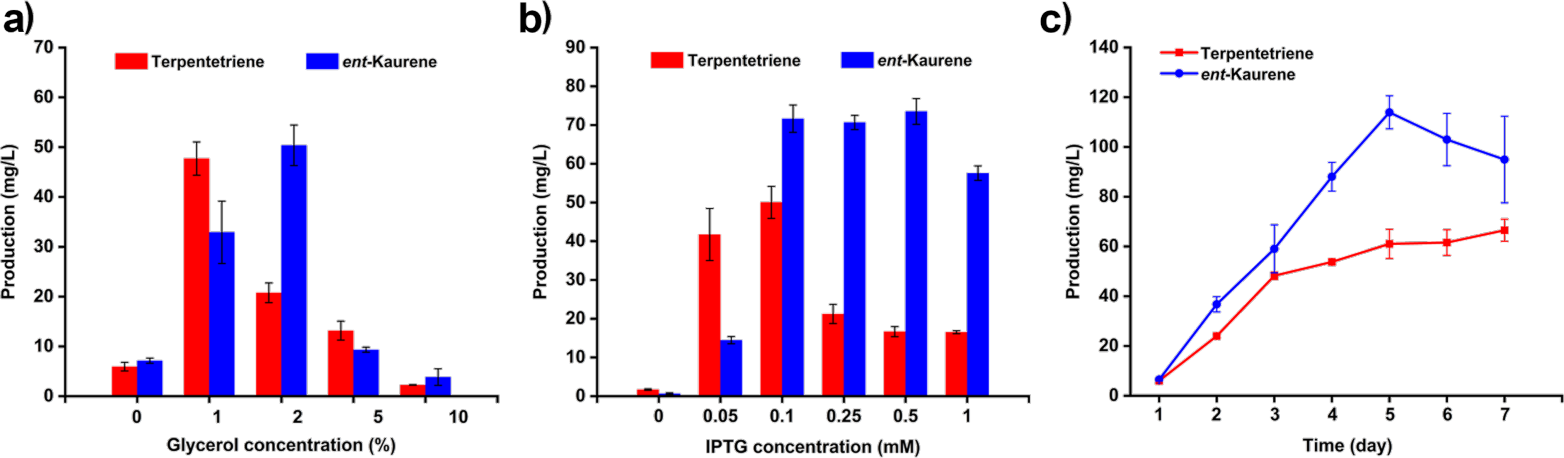
(a) Terpentetriene (red) and *ent*-kaurene (blue) yields supplied with various concentrations of glycerol. (b) Terpentetriene (red) and *ent*-kaurene (blue) yields induced with different concentrations of IPTG. (c) Time course analysis (1–7 days) of terpentetriene (red) and *ent*-kaurene (blue). All product yields are reported as means ± SD of three replicates.

Three orthogonal experiments were run to examine the effects of varying glycerol and IPTG concentrations and fermentation time, all in an effort to optimize terpentetriene and *ent*-kaurene production. Given that the carbon source is vital for overproducing natural products in *E. coli*, and glycerol is one of the most frequently used carbon sources, we first tested for optimal glycerol concentrations for terpentetriene and *ent*-kaurene production. Strains DL10004 and DL10006 were cultured in 50 mL LB medium and supplied with 0%, 1%, 2%, 5%, and 10% (v/v) glycerol, respectively. As showed in Figure 5a, the supplementary 1% (v/v) glycerol led to produce 48 ± 3 mg/L terpentetriene in DL10004, while adding 2% (v/v) glycerol resulted in the yield of 50 ± 4 mg/L *ent*-kaurene in DL10006. The production of terpentetriene and *ent*-kaurene, however, decreased significantly when more glycerol (5% or 10% (v/v)) was added, suggesting that higher glycerol concentrations might be harmful for the host cell, which was also supported by the less cell pellets harvested.

We next explored optimal IPTG concentrations (0, 0.05, 0.1, 0.25, 0.5, and 1 mM) on the production of terpentetriene and *ent*-kaurene. As shown in Figure 5b, 0.1 mM IPTG inducer led to the highest yield of terpentetriene reaching 50 ± 4 mg/L in DL10004. With an increase of IPTG concentration more than 0.1 mM, the yield of terpentetriene decreased dramatically. Additionally, although DL10006 strain could overproduce 74 ± 3 mg/L *ent*-kaurene when 0.5 mM IPTG was added, no significant decrease of the *ent*-kaurene yields under the other two IPTG concentrations (72 ± 4 mg/L for 0.1 mM and 71 ± 2 mg/L for 0.25 mM). From an economic point of view, 0.1 mM IPTG was therefore selected as the optimal concentration for both strains.

Finally, we tested the fermentation time course of DL10004 and DL10006. Based on the above established fermentation conditions, including the ratios of ISO/DMAA and the concentrations of glycerol and IPTG, we performed a sequence of parallel assays to determine the best fermentation length. Considering the inherent growth feature of *E. coli*, we set up a maximum 7-day experimental timeline for determining the titers of terpentetriene and *ent*-kaurene. As shown in Figure 5c, the yield of terpentetriene was gradually increased with the fermentation time from day 1–7, resulting in a final titer of 66 ± 4 mg/L, while the yield of *ent*-kaurene was gradually increased until day 5 before decreasing to day 7. At day 5, the titer reached 113 ± 7 mg/L for *ent*-kaurene. Taken together, the optimal fermentation conditions of both terpentetriene and *ent*-kaurene were fully established.

## Conclusion

In this study, using the truncated artificial pathways, we overproduced two clerodane and *ent*-kaurane diterpenes, terpentetriene and *ent*-kaurene, in *E. coli* by exogenously feeding ISO/DMAA to reinforce the supply of IPP and DMAPP. We optimized the ratio of ISO/DMAA, concentrations of glycerol and IPTG, and fermentation time to enhance the production of terpentetriene and *ent*-kaurene. As a result, strain DL10004, an engineered terpentetriene producer with a two-plasmid expression system, reached the titer of 66 ± 4 mg/L under the optimal conditions of supplementary 25 mM DMAA, 1% glycerol, and 0.1 mM IPTG for a 7-day shake-flask fermentation in LB medium. Strain DL0006, an engineered *ent*-kaurene producer with a two-plasmid expression system, reached the titer of 113 ± 7 mg/L under the optimal conditions of supplementary 10 mM DMAA, 2% glycerol, and 0.1 mM IPTG for a 5-day shake-flask fermentation in LB medium. Compared with the reported optimal combination of efflux pumps for *ent*-kaurene production [39], the titer was enhanced by 3.5-fold in this study. The strategy outlined here not only provides an efficient pathway to overproduce clerodane and *ent*-kaurane carbon skeletons but also offers a blueprint for coupling with emerging chemoenzymatic strategies and biocatalysis in preparation of high value diterpenoid natural products.

## Supporting Information

File 1 Experimental part and supplementary figures and tables.
