# Supplementary material for: Efficient production of clerodane and ent-kaurane diterpenes through truncated artificial pathways in Escherichia coli
Source: Beilstein J Org Chem. 2022 Jul 21;18:881–8. doi: 10.3762/bjoc.18.89 (PMC9344551; doi:10.3762/bjoc.18.89)
Supplement: File 1 — Experimental part and supplementary figures and tables. [file Beilstein_J_Org_Chem-18-881-s001.pdf]

BEILSTEIN JOURNAL OF ORGANIC CHEMISTRY

Supporting Information

for

Efficient production of clerodane and *ent*-kaurane diterpenes through truncated artificial pathways in *Escherichia coli*

Fang-Ru Li, Xiaoxu Lin, Qian Yang, Ning-Hua Tan and Liao-Bin Dong

Beilstein J. Org. Chem. **2022,** *18,* 881–888. doi:10.3762/bjoc.18.89

Experimental part and supplementary figures and tables

License and Terms: This is a supporting information file under the terms of the Creative Commons Attribution License (https://creativecommons.org/licenses/by/4.0). Please note that the reuse, redistribution and reproduction in particular requires that the author(s) and source are credited and that individual graphics may be subject to special legal provisions.
The license is subject to the *Beilstein Journal of Organic Chemistry* terms and conditions: (https://www.beilstein-journals.org/bjoc/terms)

Table of contents

Materials and methods S2–S6

Supplementary Figures S7–S10

Supplementary Tables S11–S20

References..S21

Materials and methods

General experimental procedures.

Restriction enzymes were purchased from Takara (Beijing, China). PCR primers were synthesized by Sangon Biotech Co., Ltd (Shanghai, China). DNA sequencing was performed by Sangon Biotech Co., Ltd (Shanghai, China). Phanta Super-Fidelity DNA Polymerase, ClonExpress II One Step Cloning Kit, DNA gel extraction and plasmid extraction kits were purchased from Vazyme Biotech (Nanjing, China). Other chemicals and biochemicals were purchased from standard commercial sources. ^{1}H and ^{13}C NMR experiments were run on a Bruker Avance NEO at 400 MHz for ^{1}H and 100 MHz for ^{13}C nuclei. HPLC was performed on an Agilent 1260 Infinity with an Agilent Poroshell 120 EC-C18 column (50 mm × 4.6 mm, 2.7 μm).

Plasmids construction.

The plasmids, primers, and gene sequences used in this study are listed in Tables S1, S2 and S4, respectively. Plasmids were constructed using homologous recombination [1]. The *phoN*, *ipk*, *bjks*, *citB*, and *citI* genes were codon optimized and synthesized by General biosystems (Anhui, China). The *ggdps*, *tdps*, and *ttes* genes were amplified from *Kitasatosporia griseola* DSM 43859. The plasmid pLD10001 containing *phoN* and *ipk* was synthesized by General Biosystems (Anhui, China). The *ecdps* gene was cloned from *Streptomyces sp.* NRRL S-1813. The *idi* gene was cloned from *E. coli*. Plasmid pLD10003 was constructed by inserting *ggdps* and *idi* into the *Nco*I and *Hind*III sites in the pRSFDuet-1 vector. The PCR products of *crtB* and *crtI* were assembled into the linearized vector of pRSFDuet-1 resulting in pRSFDuet-*crtB*-*crtI*. The *crtE* and *idi* genes were cloned into *Nco*I/*Bam*HI and *Nde*I/*Xho*I sites of the pRSFDuet-1 resulting in pRSFDuet-*crtE*-*idi*. The *crtB*-*crtI* and *crtE*-*idi* fragments including their T7 promoters were assembled into the linearized vector pRSFDuet-1 resulting in pLD10004. The *tdps* gene was cloned into the *Pst*I/*Hind*III sites of pRSFDuet-1 vector to construct plasmid pLD10005. Then the *ttes* gene was cloned into

the *Nde*I/*Xho*I sites of pLD10005 to construct plasmid pLD10006. After pLD10006 construction, the *tdps-ttes* fragment including their T7 promoters was cloned and inserted into pLD10003 between the *EcoR*I and *Kpn*I sites, resulting in plasmid pLD10007. The *ggdps-idi-tdps-ttes* fragment including their T7 promoters was inserted into pLD10001 between *EcoR*V and *Kpn*I sites, resulting in plasmid pLD10010. The *bjks* and *ecdps* genes were cloned into the *EcoR*V/*Xho*I and *Nde*I sites of the pRSFDuet-1 vector to construct the plasmid pLD10008. The PCR product *bjks-ecdps* including their T7 promoters was inserted into pLD10003 resulting in pLD10009. The *ggdps-bjks-ecdps-idi* fragment including their T7 promoters was inserted into pLD10001 between *EcoR*V and *Kpn*I sites, resulting in plasmid pLD10011. All plasmids were verified by DNA sequencing.

Fermentation of lycopene in engineered strains.

Each plasmid containing lycopene expression genes was transformed into *E. coli* BL21 (DE3) and plated on LB ager supplemented with ampicillin (100 μg/mL) and kanamycin (50 μg/mL) for incubation overnight at 37 °C. Colonies were picked the following day and used to inoculate 5 mL LB with ampicillin (100 μg/mL) and kanamycin (50 μg/mL) for incubation overnight at 37 °C. A 250 μL seed culture was then used to inoculate 50 mL of LB with 2% glycerol and ampicillin (100 μg/mL) and kanamycin (50 μg/mL) added and grown at 37 °C at 230 rpm. Until the culture reached $OD_{600} \approx 0.6$, flasks were cooled using an ice bath. Then, IPTG and DMAA/ISO were added to a final concentration of 0.1 mM and 6 mM, respectively. The cells were grown around 3 days at 18 °C with shaking at 200 rpm.

Identification and isolation of terpentetriene and *ent*-kaurene.

Two plasmids of pLD10001 and pLD10007 were transformed into *E. coli* BL21 (DE3) to create strain DL10004. Then, this strain was fermented in LB medium supplied with 1% glycerol and shaking at 37 °C with a speed of 230 rpm until reaching an OD_{600} of 0.6. Then, the mixture was cooled to 4 °C, followed by addition of 6 mM ISO/DMAA

3:1 and 0.1 mM isopropyl β-D-1-thiogalactopyranoside (IPTG) inducer. After a 3-day fermentation at 18 °C with a shaking speed of 200 rpm, the cells were harvested by centrifugation at 3750 rpm for 20 min at 4 °C and extracted with acetone for three times. After centrifugation, the supernatant was combined and concentrated under vacuum to obtain the crude extract, which was then passed through a silica gel column with isometric elution of pure petroleum ether to afford 45 mg terpentetriene. The pure sample was subjected to ^{1}H and ^{13}C NMR experiments.

Two plasmids of pLD10001 and pLD10009 were transformed into *E. coli* BL21 (DE3) to create strain DL10006. Then, the strain DL10006 was fermented in LB medium at 37 °C supplemented with 1% glycerol until reaching an OD_{600} of 0.6. Then, the mixture was cooled to 4 °C using an ice bath, followed by the addition of 6 mM ISO/DMAA 3:1 and 0.1 mM IPTG. After a 3-day fermentation at 18 °C with a shaking speed at 200 rpm, the cells were harvested by centrifugation at 3750 rpm for 20 min at 4 °C. The cell pellets were then extracted with acetone for three times. After centrifugation, the acetone supernatant was combined and concentrated under vacuum to obtain the crude extract, which was then passed through a silica gel column with isometric elution of pure petroleum ether to afford 90 mg *ent*-kaurene. The pure sample was subjected to ^{1}H and ^{13}C NMR experiments. Their chemical structures were determined by analysis the ^{1}H and ^{13}C NMR spectra and compared with reported data [2,3].

Terpentetriene. Colorless oil; ^{1}H NMR (400 MHz, $CDCl_3$): δ_H 6.38 (1H, dd, J = 17.6, 10.8 Hz), 5.25 (1H, d, J = 17.6 Hz), 5.16 (1H, dq, J = 2.6, 1.3 Hz), 5.06 (2H, m), 5.00 (1H, s), 2.17 (2H, td, J = 11.1, 4.7 Hz), 1.99 (3H, m), 1.65 (3H, m), 1.59 (3H, s), 1.45 (4H, m), 1.31 (1H, dq, J = 13.8, 3.4 Hz), 1.19 (1H, m), 1.06 (3H, s), 0.97 (3H, s), 0.97 (3H, d, J = 6.8 Hz) ppm; ^{13}C NMR (100 MHz, $CDCl_3$): δ_C 147.7, 144.6, 139.2, 120.3, 115.3, 113.0, 45.1, 38.3, 38.3, 37.7, 35.2, 30.2, 26.9, 25.6, 24.6, 20.6, 20.4, 18.0, 17.9, 14.9 ppm.

***ent*-Kaurene.** Needle crystal; ^{1}H NMR (400 MHz, $CDCl_3$): δ_H 4.79 (1H, s), 4.73 (1H, s), 2.64 (1H, m), 2.05 (2H, dt, J = 9.2, 2.4 Hz), 1.99 (1H, dd, J = 11.3, 2.4 Hz), 1.81 (1H, dt, J = 12.8, 3.4 Hz), 1.65 (2H, m), 1.58 (3H, m), 1.50 (4H, m), 1.36 (3H, m), 1.12 (1H, d, J = 4.5 Hz), 1.07 (1H, m), 1.02 (3H, s), 0.85 (3H, s), 1.81 (3H, s), 0.77 (2H, d, J = 2.1 Hz) ppm; ^{13}C NMR (100 MHz, $CDCl_3$): δ_C 156.2, 102.8, 56.3, 56.1, 49.2, 44.2, 44.1, 42.1, 41.3, 40.4, 39.9, 39.3, 33.7, 33.3, 33.3, 21.7, 20.3, 18.7, 18.2, 17.6 ppm.

Terpentetriene and *ent*-kaurene quantification.

The fermentation samples were extracted using equal volumes of acetone and subjected to HPLC analysis (5 μL) on an EC-C18 column by gradient elution of solvent A (H_2O + 1‰ formic acid) and solvent B (acetonitrile) with a flow rate of 0.8 mL/min over a 15 min period. The terpentetriene was subjected to HPLC analysis (λ_{max} = 210 nm) using isocratic elution (A: B = 8: 92) with a retention time at 7.7 min. The terpentetriene was subjected to HPLC analysis (λ_{max} = 202 nm) using isocratic elution (A: B = 3: 97) with a retention time at 8.3 min.

Determination of the optimal concentrations of ISO/DMAA, glycerol, and IPTG.

We performed a sequence of parallel assays including: i) supplementary pure ISO to final concentrations of 0, 2.5, 5, 10, 25, and 50 mM, respectively; ii) supplementary pure DMAA to final concentrations of 0, 2.5, 5, 10, 25, and 50 mM, respectively; and iii) supplementary ISO/DMAA 3:1 to final concentrations of 0, 2.5, 5, 10, 25, and 50 mM, respectively. Strains DL10004 and DL10006 were grown in LB medium at 37 °C and supplemented with ampicillin (100 μg/mL) and kanamycin (50 μg/mL) with a shaking speed of 230 rpm. When the OD_{600} reached ≈ 0.6, IPTG (0.1 mM) and different concentrations of ISO/DMAA listed above were added. After a 3-day fermentation at 18 °C with a shaking speed of 200 rpm, the cells were harvested by centrifugation at 3750 rpm for 20 min at 4 °C. The cell pellets were then extracted acetone for three times. After another centrifugation, the supernatant was combined and concentrated under vacuum to obtain the crude extract, which was subjected to HPLC analysis.

The procedures to determine the optimal concentrations of glycerol and IPTG were similar with that shown above except for changing the supplementary glycerol and IPTG concentrations when the OD_{600} reached ≈ 0.6.

Time course experiment of DL10004 and DL10006.

Strains DL10004 and DL10006 were grown in LB medium at 37 °C and supplemented with ampicillin (100 μg/mL) and kanamycin (50 μg/mL) with a shaking speed of 230 rpm. When the OD_{600} reached ≈ 0.6, DMAA (25 mM for terpentetriene and 10 mM for *ent*-kaurene), IPTG (0.1 mM for both terpentetriene and *ent*-kaurene), and glycerol (1% for terpentetriene and 2% for *ent*-kaurene) were added. The fermentation was then performed with shaking at 18 °C with a speed of 200 rpm. At least three flasks with 50 mL medium were harvested at the time points of day 1 to day 7. The cells were then harvested by centrifugation at 3750 rpm for 20 min at 4 °C. The cell pellets were extracted with acetone for three times. After another centrifugation, the supernatant was combined and concentrated under vacuum to obtain the crude extract, which was subjected to HPLC analysis.

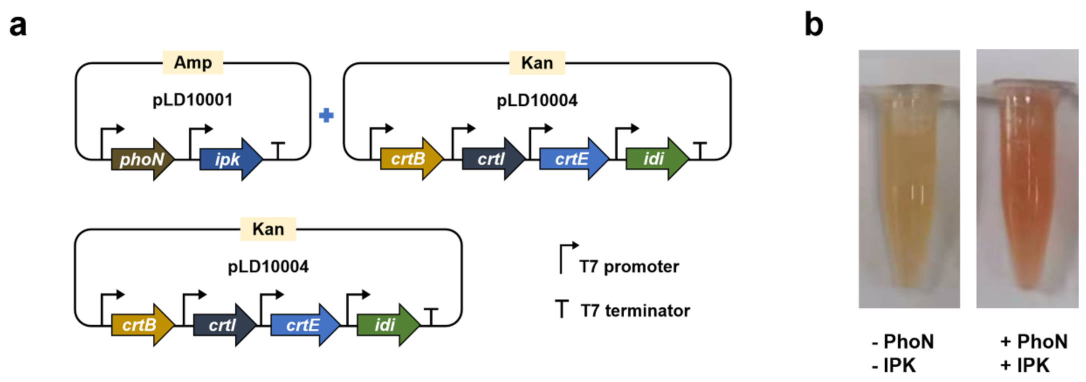

Figure S1: Truncated artificial pathways to overproduction of lycopene. (a) Plasmids design to expression of lycopene. (b) The production of lycopene was verified by the color of fermentation broth. Left: fermentation of DL10002; right: fermentation of DL10001.

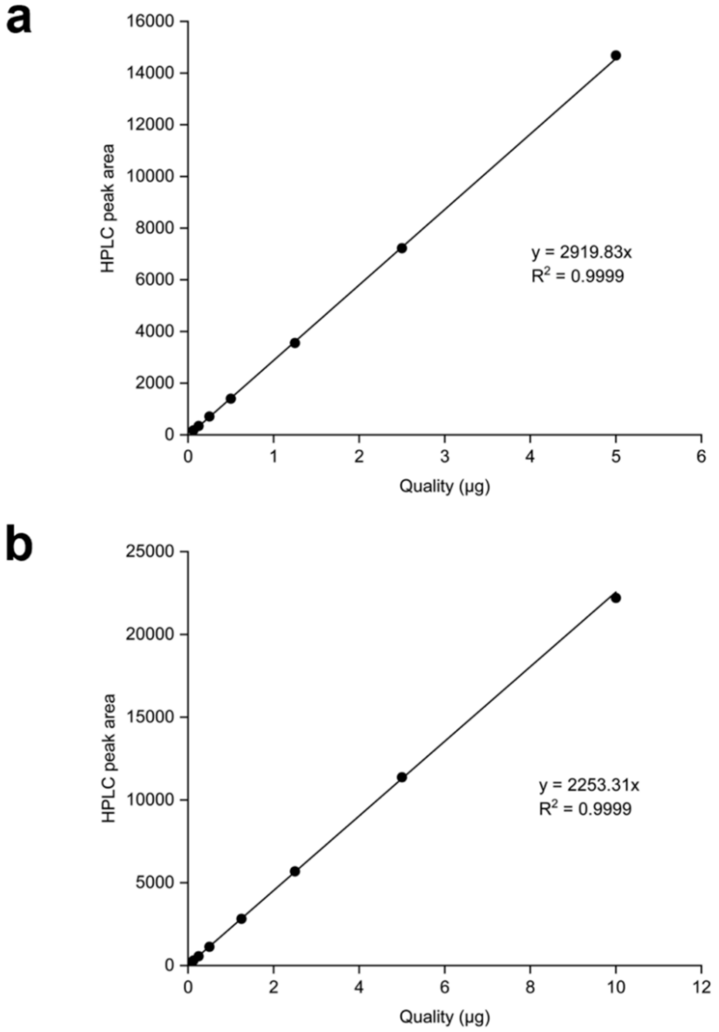

Figure S2: Quantification of terpentetriene and *ent*-kaurene. (a) Terpentetriene standard curve. (b) *ent*-Kaurene standard curve. All values represent the means ± SD of 3 replicates.

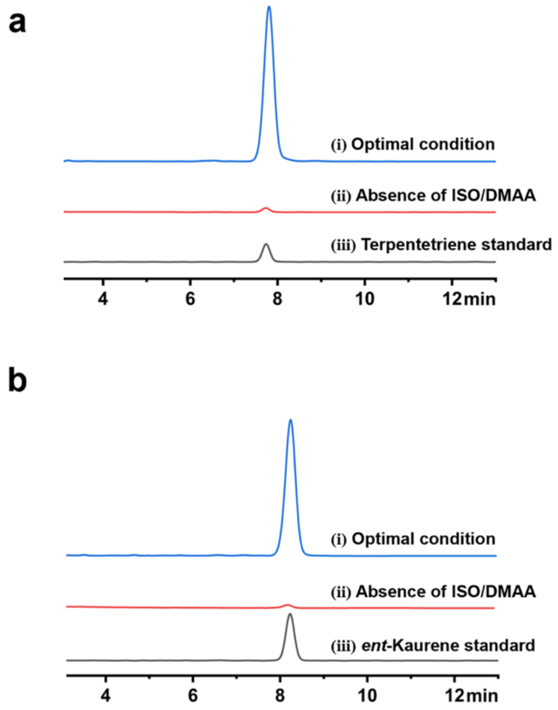

Figure S3: Terpentetriene and *ent*-kaurene were extracted in *E. coli* DL10004 and DL10006 and analyzed by HPLC. (a) The HPLC analysis of terpentetriene showed that the peak time was 7.7 min with CH_3CN/H_2O 92:8 isocratic elution. Black: standard of terpentetriene; red: fermentation conditions with 1% glycerol, 0.1 mM IPTG and absence of ISO/DMAA, fermentation for 3 days. Blue: optimal fermentation conditions with 25 mM DMAA, 1% glycerol, 0.1 mM IPTG, and fermentation for 7 days. (b) The HPLC analysis of *ent*-kaurene showed that the peak time was 8.3 min with CH_3CN/H_2O 97:3 isocratic elution. Black: standard of *ent*-kaurene; red: fermentation conditions with 1% glycerol, 0.1 mM IPTG and absence of ISO/DMAA, fermentation for 3 days. Blue: optimal fermentation conditions with 10 mM DMAA, 2% glycerol, 0.1 mM IPTG, and fermentation for 5 days.

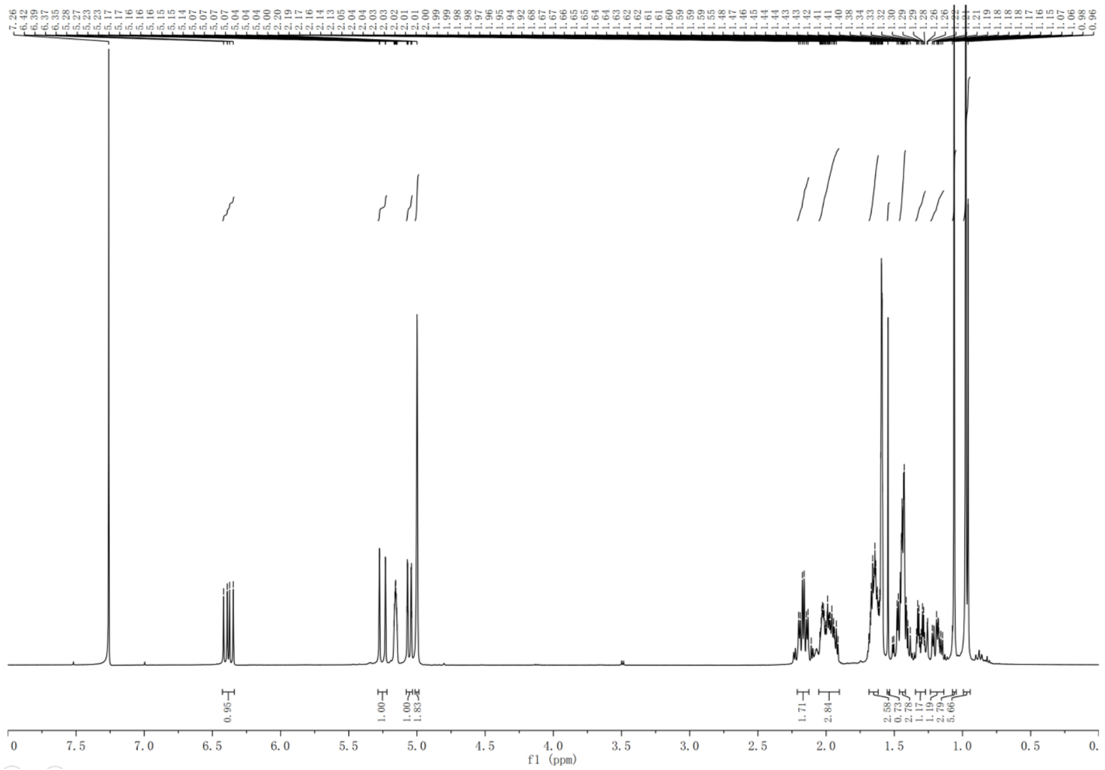

Figure S4: ^{1}H NMR spectrum of terpentetriene in $CDCl_3$ (400 MHz).

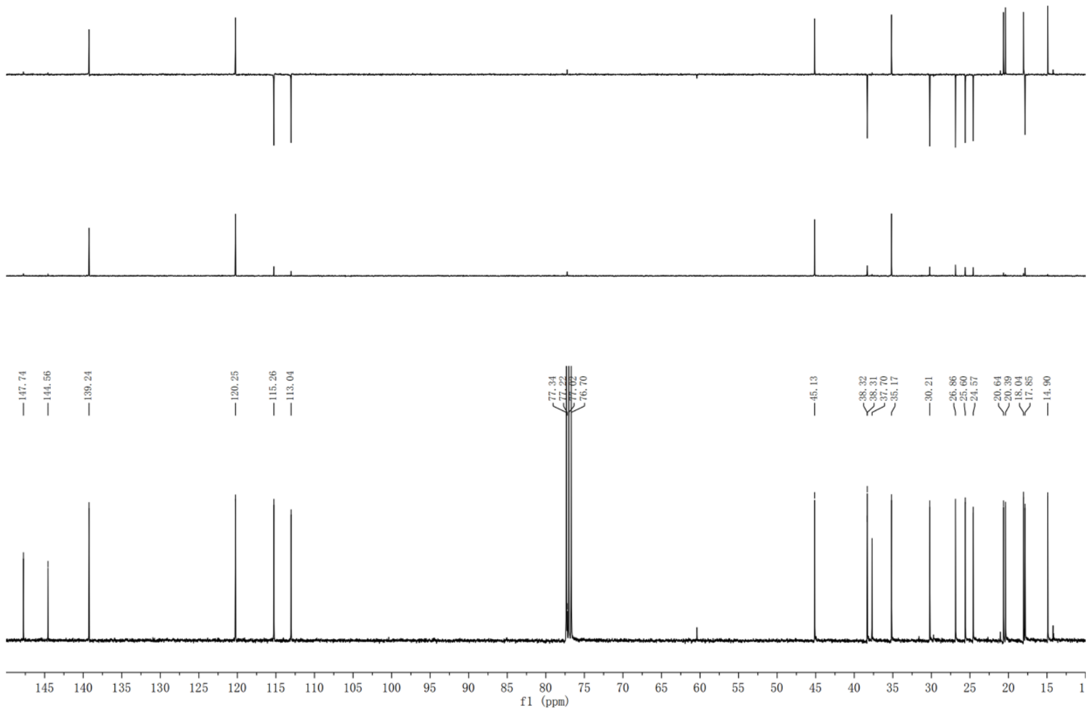

Figure S5: ^{13}C NMR spectrum of terpentetriene in $CDCl_3$ (100 MHz).

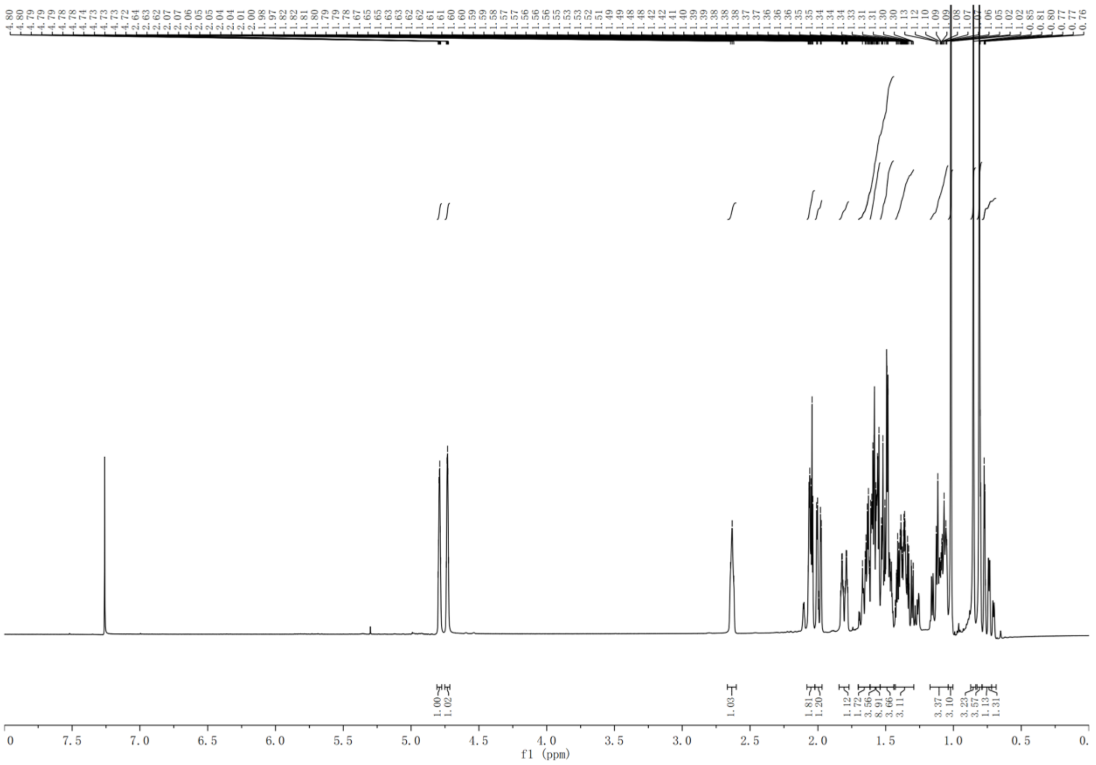

Figure S6: ^{1}H NMR spectrum of *ent*-kaurene in $CDCl_3$ (400 MHz).

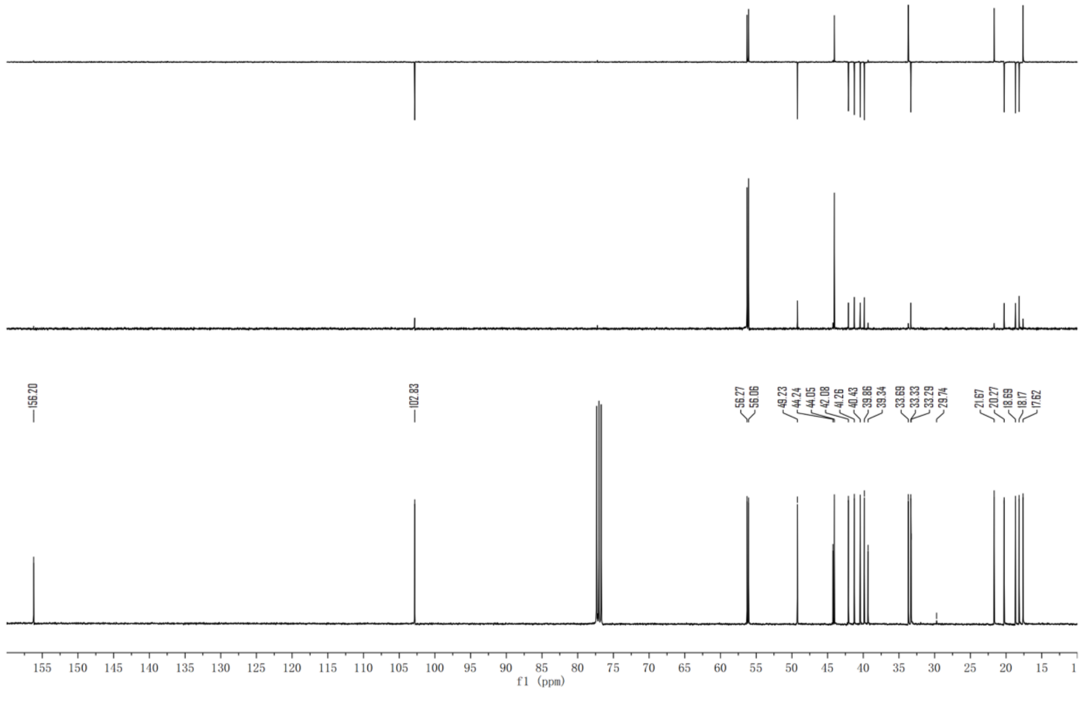

Figure S7: ^{13}C NMR spectrum of *ent*-kaurene in $CDCl_3$ (100 MHz).

Table S1: Details of primers used in this study.

Primers	Sequence 5'-3'
RSF-TDPs-F	TCATCACCACAGCCAGGATCCATGAGTGACGCCGATCGGA
RSF-TDPs-R	TCTACGCCGGACGCTCAGTACCTGCCCACGACGG
RSF-TDPs-TTE-F	AGGTACTGAGCGTCCGGCGTAGAGGAT
RSF-TDPs-TTE-R	AGGCGCGCCGAGCTCGAATTCTCAGCGGTAGCGGTTTGTCT
RSF-GGDPs-IDI-F	TAATAAGGAGATATACCATGGTGTTGTACACCGATACCGCCG
RSF-GGDPs-IDI-R	GCATTATGCGGCCGCAAGCTTTTATTTAAGCTGGGTAAATGCAGATAA
RSF-GI-TT-F	GGCAGATCTCAATTGGATAATGAGTGACGCCGATCGGA
RSF-GI-TT-R	TTTACCAGACTCGAGGGTACCTCAGCGGTAGCGGTTTGTCT
PI-GI-TT-F	GGCAGATCTCAATTGGATATCGGATCTCGACGCTCTCCCTT
PI-GI-TT-R	GCATTATGCGGCCGCAAGCTTTTATTTAAGCTGGGTAAATGCAGATAA
G-bjks-F	TCAGGAACCACTGAGGATCCGCGGCCGCATAATCGAA
G-bjks-R	TCGAGAATTCTTATGCAGGGGCGCGC
PI-GI-eCDPs-bjks-F	GGCAGATCTCAATTGGATATCGGATCTCGACGCTCTCCCTT
PI-GI-eCDPs-bjks-R	TTTACCAGACTCGAGGGTACCTTATTTAAGCTGGGTAAATGCAGATAA
pRSF-CrtB-F	TAATAAGGAGATATACCATGGTGAACAACCCGTCTCTGCTGA
pRSF-CrtB-R	TTACTTTCTGTTCGACTTAAGTCATCACAGCGGACGCTGC
pRSF-CrtI-F	TAAGAAGGAGATATACATATGAAACCGACCACCGTTATCGG
pRSF-CrtI-R	CGTCGAGATCCTCATCAGATCAGGTCTTCCAGCA
BI-EI-F	ATCTGATGAGGATCTCGACGCTCTCCCTT
BI-EI-R	GGTTTCTTTACCAGACTCGAGTTATTTAAGCTGGGTAAATGCAGATAA

Table S2: Plasmids used in this study.

Plasmid	Description	Reference
pETDuet-1	Plasmid for cloning, Amp ^{r}	Novagen
pRSFDuet-1	Plasmid for cloning, Kan ^{r}	Novagen
pLD10001	pETDuet-1 harboring *phoN* and *ipk,* Amp^r	Synthesized
pLD10002	pRSFDuet-1 harboring *ggdps*, Kan ^{r}	This study
pLD10003	pRSFDuet-1 harboring *ggdps* and *idi*, Kan ^{r}	This study
pLD10004	pRSFDuet-1 harboring *crtB*, *crtI*, *crtE* and *idi*, Kan ^{r}	This study
pLD10005	pRSFDuet-1 harboring *tdps*, Kan ^{r}	This study
pLD10006	pRSFDuet-1 harboring *tdps* and *ttes*, Kan ^{r}	This study
pLD10007	pRSFDuet-1 harboring *ggdps*, *idi*, *tdps* and *ttes*, Kan ^{r}	This study
pLD10008	pRSFDuet-1 harboring *ecdps* and *bjks*, Kan ^{r}	This study
pLD10009	pRSFDuet-1 harboring *ggdps*, *bjks*, *ecdps* and *idi*, Kan ^{r}	This study
pLD10010	pLD10001 harbing *ggdps*, *idi*, *tdps* and *ttes*, Amp ^{r}	This study
pLD10011	pLD10001 harbing *ggdps*, *idi*, *ecdps* and *bjks*, Amp ^{r}	This study

Table S3: Strains used in this study.

Strain	Genotype, Description	Reference
E. coli DH5α	*E. coli* host for general cloning	General biosystems (Anhui, China)
E. coli BL21 (DE3)	*E. coli* host for protein expression	General biosystems (Anhui, China)
Kitasatosporia griseola DSM 43859	*Streptomyces* used to extract the genome as a PCR template	CGMCC
Streptomyces sp. NRRL S-1813	*Streptomyces* used to extract the genome as a PCR template	This study
DL10001	*E. coli* BL21 containing pLD10001 and pLD10004	This study
DL10002	*E. coli* BL21 containing pLD10004	This study
DL10003	*E. coli* BL21 harboring pLD10010	This study
DL10004	*E. coli* BL21 harboring pLD10001 and pLD10007	This study
DL10005	*E. coli* BL21 harboring pLD10011	This study
DL10006	*E. coli* BL21 harboring pLD10001 and pLD10009	This study

Table S4: Gene sequences of proteins used in this study.

Protein	Sequence (5’ →3’)
ipk from *Thermoplasma acidophilum* codon-optimized for *E. coli*	ATGGACCCTTTTACCATGATGATTCTGAAAATTGGCGGCAG<br>CGTTATTACCGATAAAAGTGCATATCGTACCGCACGTACCT<br>ATGCCATTCGTAGCATTGTTAAAGTGCTGAGTGGTATTGAA<br>GATCTGGTGTGTGTTGTTCATGGTGGCGGTAGCTTTGGTCAT<br>ATTAAGGCAATGGAATTTGGCCTGCCGGGTCCGAAAAATCC<br>GCGTAGTAGTATTGGTTATAGTATTGTTCATCGCGATATGGA<br>AAATCTGGATCTGATGGTTATTGATGCAATGATTGAAATGG<br>GTATGCGCCCGATTAGCGTGCCGATTAGCGCACTGCGCTAT<br>GATGGTCGTTTTGATTATACCCCGCTGATTCGTTATATTGAT<br>GCAGGCTTTGTGCCGGTTAGCTATGGTGACGTTTATATTAAG<br>GATGAACATAGCTATGGTATCTATAGTGGTGACGATATTAT<br>GGCAGATATGGCAGAACTGCTGAAACCGGATGTGGCCGTTT<br>TTCTGACCGATGTGGATGGCATCTATAGTAAAGATCCGAAA<br>CGCAATCCGGATGCAGTGCTGCTGCGCGATATTGATACCAA<br>TATTACCTTTGATCGCGTGCAGAATGATGTTACCGGTGGCAT<br>TGGTAAAAAAATTTGAAAGTATGGTGAAGATGAAGAGTAGTG<br>TGAAAAATGGTGTGTATCTGATTAATGGTAACCATCCGGAA<br>CGTATTGGTGACATTGGCAAAGAAAGTTTTATTGGCACCGT<br>TATTCGTTAA
phoN from *Shigella flexneri* codon-optimized for *E. coli*	ATGAAGCGCCAGCTGTTTACCCTGAGCATTGTGGGTGTTTTT<br>AGTCTGAATACCTTTGCCAGTATTCCGCCGGGTAATGATGTT<br>ACCACCAAACCGGATCTGTATTATCTGACCAATGATAATGC<br>AATTGACAGCCTGGCCCTGCTGCCGCCGCCGCCTCAGATTG<br>GTAGCATTGCATTTCTGAATGATCAGGCAATGTATGAAAAA<br>GGTCGCCTGCTGCGTAATACCGAACGTGGTAAACTGGCCGC<br>CGAAGATGCAAATCTGAGCAGTGGCGGTGTTGCAAATGTGT<br>TTAGCGCCGCCTTTGGTAGCCCGATTACCGCCAAAGATAGC<br>CCGGAACTGCATAAACTGCTGACCAATATGATTGAAGATGC<br>AGGCGATCTGGCAACCCGCAGCGCAAAAGAATATTATATGC<br>GTATTCGTCCGTTTGCCTTTTATGGTGTTAGTACCTGTAATA<br>CCAAAGAACAGGATACCCTGAGCCGTAATGGCAGTTATCCG<br>AGTGGCCATACCAGCATTGGTTGGGCCACCGCACTGGTTCT<br>GAGTGAAATTAATCCGGCCCGCCAGGATACCATTCTGAAAC<br>GTGGCTATGAACTGGGTGACAGTCGTGTGATTTGCGGTTAT<br>CATTGGCAGAGTGATGTGGATGCAGCACGTATTGTGGGTAG<br>TGCCATTGTGGCAACCCTGCATAGCAATCCGGTGTTTCAGG<br>CCCAGCTGCAGAAAGCAAAAGATGAATTTGCCAATAATCAG<br>AAAAAGTAA
crtB from *Pantoea ananatis*	ATGAACAACCCGTCTCTGCTGAACCACGCGGTTGAAACCAT<br>GCCGGTTGGTTCTAAATCTTTCGCGACCGCGTCTAAACTGTT<br>CGACGCGAAAACCCGTCGTTCTGTTCTGATGCTGTACGCGT

was codon-optimized for *E. coli*	GGTGCCGTCACTGCGACGACGTTATCGACGACCAGACCCTG<br>GGTTTCCAGGCGCGTCAGCCGGCGCTGCAGACCCCGGAACA<br>GCGTCTGATGCAGCTGGAAATGAAAACCCGTCAGGCGTACG<br>CGGGTTCTCAGATGCACGAACCGGCGTTCGCGGCGTTCCAG<br>GAAGTTGCGATGGCGCACGACATCGCGCCGGCGTACGCGTT<br>CGACCACCTGGAAGGTTTCGCGATGGACGTTCGTGAAGCGC<br>AGTACTCTCAGCTGGACGACACCCTGCGTTACTGCTACCAC<br>GTTGCGGGTGTTGTTGGTCTGATGATGGCGCAGATCATGGG<br>TGTTCGTGACAACGCGACCCTGGACCGTGCGTGCGACCTGG<br>GTCTGGCGTTCCAGCTGACCAACATCGCGCGTGACATCGTT<br>GACGACGCGCACGCGGGTCGTTGCTACCTGCCGGCGTCTTG<br>GCTGGAACACGAAGGTCTGAACAAAGAAAACTACGCGGCG<br>CCGGAAAACCGTCAGGCGCTGTCTCGTATCGCGCGTCGTCT<br>GGTTCAGGAAGCGGAACCGTACTACCTGTCTGCGACCGCGG<br>GTCTGGCGGGTCTGCCGCTGCGTTCTGCGTGGGCGATCGCG<br>ACCGCGAAACAGGTTTACCGTAAAATCGGTGTTAAAGTTGA<br>ACAGGCGGGTCAGCAGGCGTGGGACCAGCGTCAGTCTACCA<br>CCACCCCGGAAAAACTGACCCTGCTGCTGGCGGCGTCTGGT<br>CAGGCGCTGACCTCTCGTATGCGTGCGCACCCGCCGCGTCC<br>GGCGCACCTGTGGCAGCGTCCGCTGTGATGA
crtI from *Pantoea ananatis* was codon-optimized for *E. coli*	ATGAAACCGACCACCGTTATCGGTGCGGGTTTCGGTGGTCT<br>GGCGCTGGCGATCCGTCTGCAGGCGGCGGGTATCCCGGTTC<br>TGCTGCTGGAACAGCGTGACAAACCGGGTGGTCGTGCGTAC<br>GTTTACGAAGACCAGGGTTTCACCTTCGACGCGGGTCCGAC<br>CGTTATCACCGACCCGTCTGCGATCGAAGAACTGTTCGCGC<br>TGGCGGGTAAACAGCTGAAAGAATACGTTGAACTGCTGCCG<br>GTTACCCCGTTCTACCGTCTGTGCTGGGAATCTGGTAAAGTT<br>TTCAACTACGACAACGACCAGACCCGTCTGGAAGCGCAGAT<br>CCAGCAGTTCAACCCGCGTGACGTTGAAGGTTACCGTCAGT<br>TCCTGGACTACTCTCGTGCGGTTTTCAAAGAAGGTTACCTGA<br>AACTGGGTACCGTTCCGTTCCTGTCTTTCCGTGACATGCTGC<br>GTGCGGCGCCGCAGCTGGCGAAACTGCAGGCGTGGCGTTCT<br>GTTTACTCTAAAGTTGCGTCTTACATCGAAGACGAACACCT<br>GCGTCAGGCGTTCTCTTTCCACTCTCTGCTGGTTGGTGGTAA<br>CCCGTTCGCGACCTCTTCTATCTACACCCTGATCCACGCGCT<br>GGAACGTGAATGGGGTGTTTGGTTCCCGCGTGGTGGTACCG<br>GTGCGCTGGTTCAGGGTATGATCAAACTGTTCCAGGACCTG<br>GGTGGTGAAGTTGTTCTGAACGCGCGTGTTTCTCACATGGA<br>AACCACCGGTAACAAAATCGAAGCGGTTCACCTGGAAGAC<br>GGTCGTCGTTTCCTGACCCAGGCGGTTGCGTCTAACGCGGA<br>CGTTGTTCACACCTACCGTGACCTGCTGTCTCAGCACCCGGC<br>GGCGGTTAAACAGTCTAACAAACTGCAGACCAAACGTATGT<br>CTAACTCTCTGTTCGTTCTGTACTTCGGTCTGAACCACCACC<br>ACGACCAGCTGGCGCACCACACCGTTTGCTTCGGTCCGCGT

	TACCGTGAACTGATCGACGAAATCTTCAACCACGACGGTCT<br>GGCGGAAGACTTCTCTCTGTACCTGCACGCGCCGTGCGTTA<br>CCGACTCTTCTCTGGCGCCGGAAGGTTGCGGTTCTTACTACG<br>TTCTGGCGCCGGTTCCGCACCTGGGTACCGCGAACCTGGAC<br>TGGACCGTTGAAGGTCCGAAACTGCGTGACCGTATCTTCGC<br>GTACCTGGAACAGCACTACATGCCGGGTCTGCGTTCTCAGC<br>TGGTTACCCACCGTATGTTCACCCCGTTCGACTTCCGTGACC<br>AGCTGAACGCGTACCACGGTTCTGCGTTCTCTGTTGAACCG<br>GTTCTGACCCAGTCTGCGTGGTTCCGTCCGCACAACCGTGAC<br>AAAACCATCACCAACCTGTACCTGGTTGGTGCGGGTACCCA<br>CCCGGGTGCGGGTATCCCGGGTGTTATCGGTTCTGCGAAAG<br>CGACCGCGGGTCTGATGCTGGAAGACCTGATCTGATGA
bjks from *Bradyrhizobium japonicum* codon-optimized for *E. coli*	ATGATCCAGACCGAACGCGCAGTGCAGCAGGTTCTGGAATG<br>GGGTCGCAGCCTGACCGGCTTTGCAGATGAACATGCCGTGG<br>AAGCAGTTCGCGGTGGTCAGTATATTCTGCAGCGCATTCAT<br>CCGAGCCTGCGTGGTACCAGCGCACGTACCGGTCGTGATCC<br>GCAGGATGAAACCCTGATTGTGACCTTTTATCGTGAACTGG<br>CCCTGCTGTTTTGGCTGGATGATTGTAATGATCTGGGTCTGA<br>TTAGCCCGGAACAGCTGGCAGCCGTTGAACAGGCACTGGGC<br>CAGGGCGTGCCGTGCGCATTACCGGGTTTTGAAGGTTGTGC<br>CGTGCTGCGTGCAAGTCTGGCCACCCTGGCATATGATCGCC<br>GCGATTATGCACAGCTGCTGGATGATACCCGTTGTTATAGT<br>GCAGCACTGCGCGCAGGTCATGCCCAGGCAGTTGCCGCCGA<br>ACGCTGGAGTTATGCAGAATATCTGCATAATGGCATTGATA<br>GCATTGCCTATGCAAATGTTTTCTGTTGTCTGAGTCTGCTGT<br>GGGGTCTGGATATGGCAACCCTGCGCGCACGCCCGGCATTT<br>CGTCAGGTTCTGCGCCTGATTAGTGCAATTGGCCGTCTGCAG<br>AATGATCTGCATGGTTGTGATAAAGATCGTAGCGCCGGCGA<br>AGCAGATAATGCCGTTATTCTGCTGCTGCAGCGCTATCCGG<br>CCATGCCGGTGGTTGAATTTCTGAATGATGAACTGGCAGGT<br>CATACCCGTATGCTGCATCGCGTGATGGCAGAAGAACGTTT<br>TCCGGCCCCGTGGGGTCCGCTGATTGAAGCAATGGCAGCAA<br>TTCGCGTGCAGTATTATCGTACCAGCACCAGTCGCTATCGCA<br>GCGATGCAGTGCGTGGTGGCCAGCGCGCCCCTGCATAA
idi from *E. coli* DH5α	ATGCAAACGGAACACGTCATTTTATTGAATGCACAGGGAGT<br>TCCCACGGGTACGCTGGAAAAGTATGCCGCACACACGGCAG<br>ACACCCGCTTACATCTCGCGTTCTCCAGTTGGCTGTTTAATG<br>CCAAAGGACAATTATTAGTTACCCGCCGCGCACTGAGCAAA<br>AAAGCATGGCCTGGCGTGTGGACTAACTCGGTTTGTGGGCA<br>CCCACAACTGGGAGAAAGCAACGAAGACGCAGTGATCCGC<br>CGTTGCCGTTATGAGCTTGGCGTGGAAATTACGCCTCCTGA<br>ATCTATCTATCCTGACTTTCGCTACCGCGCCACCGATCCGAG<br>TGGCATTGTGGAAAATGAAGTGTGTCCGGTATTTGCCGCAC<br>GCACCACTAGTGCGTTACAGATCAATGATGATGAAGTGATG

	GATTATCAATGGTGTGATTTAGCAGATGTATTACACGGTATTGATGCCACGCCGTGGGCGTTCAGTCCGTGGATGGTGATGCAGGCGACAAATCGCGAAGCCAGAAAACGATTATCTGCATTTACCCAGCTTAAATAA
ecdps from *Streptomyces sp.* NRRL S-1813	ATGCTCGAAGTTCCCGCTCAGCCCACGCCCGCCCCCCGCGAGGCCGAGGCGGCCGCGCTGCTCGCGGCGACCGTCCACGACCCCTGGGGCCTGGTCGCTCCGTCGGTGTACGACACCGCCCGGCTGGTCTCCCTCGCCCCGTGGCTCGACGGCCACCGGGAGCGTCTCGGCTATCTGGTCGAGGAGCAGAACCAGGACGGAAGCTGGGGCGCACCCGACGGGTACGGCCTGGTACCCACGCTCAGTGCGGTGGAGGCGCTGCTGACCGAACTCGCCCGGCCGGAATCCGGCGCGCCGCACCCGCCCCACGACGACCTCGCCGCGGCCTGCGCCGGCGGTCTGGGCGCCCTCCAGGACGGTCTGCTCGCCGGTCCGGTGCCCGACACCATCGGCGTCGAGTTCGTCGCGCCGTCCCTGCTCGCGGACATCAACACCCGGCTGGCCGCGCTGACCGAGCAGGCACCCGGCAAGCTCGGGGCATGGTCCGGCACCACCCTGACGTCACCGGCGCCCGACCTGGACGGTGCGCTGCTGGCCGGCGTCCGGGAGATGACCGAGCAGGCGCCGCTGCCGGAGAAGCTGTGGCACACACTGGAGGCCATCACCCGCGACGGCACCCGCGGTGCCCGGCCGCACGAGGGCGCACCGCCGCACAACGGCTCGGTCGGCTGCTCCCCCGCCGCCACCGCCGCCTGGCTGGGCGCCTCGCCCGATCCGGCCGCGCCGGGCGTCGCCTATCTCCGTGACGTCCAGGCGCGGTTCGGCGGGCCGGTGCCCTCGATCACCCCGATCGTCTACTTCGAGCAGGCGTGGGTCCTCAACTCGCTGGCCGCCTCCGGCCTGCGCTACGAGGCCCCGGCCGCGCTCCTCGACAGCCTCGAAGCGGGTCTCACGGACGAGGGCACAGCCGCCGCCCCCGGTCTGCCGAGCGACTCCGACGACACCGCCGCCGTCCTCTTCGCCCTGGCGCAGCACGGCAGGACGCACCGCCCCGACAGCCTGATGCACTTCCGCCGGGACGGCTACTTCTCCTGCTTCGGCGTCGAGCGCACCCCCTCCACCAGCACCAACGCACACATCCTGGAGGCCCTCGGCCATCACGTCACGGTGCGCCCCGACGACGCGGGACGCTATGGCGCGGAGATCCGGATGATCAGCGACTGGCTGCTGGACAACCAGCTGCCCGACGGCAGCTGGATGGACAAGTGGCACGCCTCGCCGTACTACGCCACGGCCTGCTGTGCGCTGGCGCTCGCCGAGTTCGGCGGCCCGTCCGCACGGGCCGCGGTCGGCCGGGCCGCCGCGTGGGCACTGGCGACCCAGCGCGCCGACGGCTCCTGGGGACGCTGGCAGGGCACCACGGAGGAGACCGCGTACATGGTGCAGCTCCTGATGCGTACCCGTACCCCCGGGAGCCCGGGGACCGTCGCCCGGTCGGCGGCCCGCGGCTGCGACGCGCTGCTGGCCCACGACGACCCGGCCTCCTACCCCGGGCTCTGGCACGACAAGGACATCTACGCGCCGGTGACCGTCATCCGGGCGGCGCGGCTCGCGGCACTGGCGCTCGGCGGCGCCGAGTCCGCCGCTTCCGGAGGTGCTTGA

ggdps from *Kitasatospora griseola* DSM 43859	ttgtacaccgataccgccgaaacactggagagcgcagtcgatgcgggccgcaaccacaccttgcg<br>ggcagccggaatcgccacacctgcactgcgcgccacgattgagacgctcggcagccgaatacgc<br>ccgatcgtcggttaccacttcggctggctggacctggcggggcagccgacggaccagggcagcg<br>gcaagatgattcgcgcggccctgaccgtcctcgcggcggaggcctgcgggggcgatgtccagcg<br>ggcggtgtgtggcgccgtcgcggtcgagttggtgcacaacttctccctgctccacgacgacgtcat<br>ggacggtgatctcaagcgcagaggacgccccactgtctgggccacgttgggtgtcccggccgcg<br>atcctggccggcgacgttctgctcgcgcgtggctgcgcgatcctggagggcgactccgagcaccg<br>gacctgggccaccaagctgctcaccgagacgatcgcgacgctcgccgaagggcagatggcgga<br>tgtggcgttcgagcaccgcacgaccatctcgctggaggaggcgctggccgtctccgaggcgaag<br>accgccgcgctgctgagctgcgcctgcgagctgggtgcgggactctccgggggcggggccgag<br>gctcgtgagcgcctggccaggttcggatggcatctggggatggcgttccagctggtggatgacgtc<br>ctgggcatctggggcgaccccgcggccaccggcaagccggccggttccgatctgcggaacagg<br>aagaagagcatcccggtggccgcggcgatgaacggcaccggcgcagccgccgaggagttggc<br>cgcgatctaccgcggcgcggagccgctgaccgacgagatggccgaccgcgccgccaagctgat<br>cgagtcggccggcggacgcgcgtggacggaagccgagatcgaccggcacaggaggggtgcg<br>gcggatcagctggaggctttgggcctcaccgaggagcaacgccggccgctgctggcgatggccg<br>actatgtcgctttcaggaaccactga
tdps from *Kitasatospora griseola* DSM 43859	atgagtgacgccgatcggatagcggccctgttgaaggaccgcgctgccgacccggtaacgaagtt<br>cagcccttcgccgtacgagaccggacagttcctgcggatatccgagcgtgcggacgtgggcacac<br>cgcagatcgactacttgctcgcgacgcagcgaccggacggcctgtgggggtcggtcgggttcga<br>gctcgtaccgacgctgggcgcggtggcgggtctgtcctcgcggccggaatacgccgaccgggcc<br>ggagtgacggacgctgtggcgcgggcctgcgaaaagctctgggagctggcgctcggtgagggc<br>gggctgccgaggctgcccgacaccgtggcatccgagatcatcgtgcccagcctgatcgatctgct<br>gagcgaggtgctgcagcgccaccgcccggctgcagccggcaaggccgggcacgagcaggagt<br>ttcccggtccgccgggcgcgaagcctgagctgtggcgccagttgagcgaccggatcgcccaggg<br>gcaggccatcccgaagacggcatggcacacacttgaggcgttccatccgctgccgaaacagttcg<br>cggcgacggtaacgcccgccgcggacggtgcggtgacgtgttctccgtcgtccacagcggcctg<br>ggtgtcggctgtggggacggacgccggggcatcgactcgcgcctatctcgatgaagcgcagagc<br>cgctacggcggagcgatcccgatgggaagctccatgccgtacttcgaggtgctctgggtgctcaac<br>ctggtgctcaagtacttcccggacgtgccgatccccagggaaatcatcgaagagattgccgccgga<br>ttcagcgattcgggcatcggtggcgggccgggtctgccgcccgacggagacgacacggcctacg<br>ccaatctcgccggcgacaaactcggcgcccccactcacccggaaatcctgatgaagttctgggcc<br>gaggaccacttcgtttcgtatccggcggagcaaacgccctcggagaccgtgaacgcacacgccct<br>cgagtacctcaatcatctcaggatgcgccgtggcatcacggaatacggtgccatcgaggacgcatg<br>tgccgagtgggtgatctcccagcagaccgaggacggctgctggtacgacaaatggaatgtctcgc<br>cgtactattccacggcggcatgtgtcgaagccctgctcgacgcccggaagcaggatgaaccgcag<br>ctggattcgctgcgtcgcgccagggaatggctgcttcggcatcagacggattcgggcggctgggg<br>aatggcggagccgtcacccgaggaaacggcctacgcggtgctggcgctcgacctgttcgcgagc<br>catggcggcgagggcgccgaggagtgtgcggcagcgatatccagggccaaggacttcttcaagg<br>acgagtccagggagaatcccccgctgtggatgggcaaggacctgtacacgcctttccggatcgtc<br>gacgtgacggtgatgtgcggccgtgccgtcgtgggcaggtactga
ttes from *Kitasatospora*	atgcccgacgcgatcgagttcgagcacgaaggccggcggaacccgaattcggcggaggccgaa<br>tcggcctacagctccatcatcgctgcgctcgaactccaggaatccgactacgccgtgatttccgggc<br>actcgaggatcgtgggggccgcggcgctggtctatcccgatgcggatgccgaaaccttgctggcg

griseola DSM 43859	gcttcgctgtggacggcctgcctcatcgtcaacgacgaccggtgggactacgtccaggaggacggcgggcggctggctccgggcgagtggttcgacggggtcaccgaggtcgtggacacctggcgaactgccgggccccgtctgtcggaccccttcttcgagctggtacggacaacgatgtcccggctcgacgcagcgctcggcgcggaagcagcggacgagatcggccacgaaatcaagcgcgccatcacggcgatgaagtgggaaggggtatggaacgaatacaccaagaagacgtccttggcgacgtatctgagcttccgccgcggctactgcacgatggacgtccaggtcgttctggacaagtggatcaacggcggccgcagtttcgcagcgctgcgtgacgaccccgtccgacgagcgatcgacgatgtggtggtgcggttcggctgtctgtcgaacgactactactcgtggggccgggagaagaaggcggtcgacaagtcgaatgcggtgcggatcctgatggaccacgccggctacgacgagagcaccgcgctggcccacgttcgcgacgactgcgtgcaggcgatcaccgacctggactgcatcgaggaatccatcaagcgcagcggccatctcggcagccatgcacaggagttgctcgactacctcgcgtgccatcgaccgctgatctacgcagcggcgacctggccgacggagacaaaccgctaccgctga

Table S5: Abbreviations commonly used in this study.

Title	Abbreviation
isopentenyl diphosphate	IPP
dimethylallyl diphosphate	DMAPP
mevalonate	MVA
methylerythritol phosphate	MEP
geranylgeranyl diphosphate synthases	GGDPs
ent-copalyl diphosphate synthases	*e*CDPs
terpentedienol diphosphate synthase	TDPs
terpentetriene synthase	TTEs
polymerase chain reaction	PCR

References

1. Pines, G.; Freed, E. F.; Winkler, J. D.; Gill, R. T. *ACS Synth. Biol.* **2015,** *4*, 1176-1185. doi:10.1021/acssynbio.5b00009
2. Dairi, T.; Hamano, Y.; Kuzuyama, T.; Itoh, N.; Furihata, K.; Seto, H. *J. Bacteriol.* **2001,** *183*, 6085-6094. doi:10.1128/JB.183.20.6085-6094.2001
3. Liu, W.; Feng, X.; Zheng, Y.; Huang, C.-H.; Nakano, C.; Hoshino, T.; Bogue, S.; Ko, T.-P.; Chen, C.-C.; Cui, Y.; Li, J.; Wang, I.; Hsu, S.-T. D.; Oldfield, E.; Guo, R.-T. *Sci. Rep.* **2014,** *4*, 6214. doi:10.1038/srep06214
